# Editorial: Vein of galen malformation: a scientific and clinical journey targeting the best outcome

**DOI:** 10.3389/fped.2023.1323889

**Published:** 2023-11-03

**Authors:** Silvia Buratti, Darren B. Orbach, Prakash Muthusami, Fergus Robertson

**Affiliations:** ^1^ Neonatal and Pediatric Intensive Care Unit, Acceptance and Emergency Department, IRCCS Istituto Giannina Gaslini, Genoa, Italy; ^2^ Neurointerventional Radiology, Boston Children’s Hospital, Boston, MA, United States; ^3^ Interventional Radiology, University of Toronto and the Hospital for Sick Children, Toronto, ON, Canada; ^4^ Department of Radiology, Great Ormond Street Hospital, London, United Kingdom

**Keywords:** VGAM, children, research, outcome, treatment

**Editorial on the Research Topic** Vein of galen malformation: a scientific and clinical journey targeting the best outcome

Vein of Galen aneurysmal malformation (VGAM) represents nearly 30% of all pediatric cerebrovascular malformations, yet, as a rare condition, presents several knowledge gaps that should be addressed. Over the past three decades, advances in diagnostic and therapeutic strategies have resulted in markedly improved survival and prognosis, but mortality and adverse neurological outcomes are unfortunately still not rare.

When we initially proposed the specific topics for this editorial assignment, we included all aspects of research that could structure a scientific and clinical journey focusing on the best outcome (Table 1). The contributions to this project highlight the extraordinary interest that this condition arouses in the multidisciplinary group of specialists involved in the care of children with VGAM, and through the submitted manuscripts several aspects have been studied and discussed from diverse perspectives.

**Table 1:** Vein of galen malformation: a scientific and clinical journey targeting the best outcome. Research topics.

VGAM research topics
• Defining genetic background of VGAM
• Embryonic considerations and anatomical features
• Prenatal assessment and prognostic factors
• Pathological features: from brain damage to lung and heart pathological changes secondary to AV shunt and overflow
• Neuroradiological aspects and identification of prognostic factors
• Interventional radiology: treatment challenges
• In utero VGAM treatment
• Intensive care management
• Multidisciplinary long term follow-up and outcomes
• A challenging project: the VGAM International Registry

The genetic background and the molecular pathophysiology of cerebrovascular malformations represent a key topic in research and several genes encoding proteins involved in vascular development have been identified in association with VGAM (1). Tas et al. in the retrospective review “Arteriovenous Cerebral High Flow Shunts in Children: From Genotype to Phenotype” observed that Ephrin type-B receptor 4 *(EPHB4)* variants seemed to be specific to VGAM, RASp21 Protein Activator 1 (*RASA1)* variants were associated with either pial arteriovenous fistulas or with VGAM, and Hereditary Hemorrhagic Telangiectasia (HHT) gene variants seemed specific to pial arteriovenous fistulas not draining into the vein of Galen. As stated by the authors, misclassifications of AV fistulas or malformations that drain into a dilated vein of Galen as true VGAMs are frequent and may lead to catastrophic consequences if deep venous drainage is not well defined. This is a key point in treatment strategies and in the prevention of severe hemorrhagic or ischemic procedural complications, especially at the earliest ages, when it is difficult to definitively establish a clear diagnosis and classification.

Since the late 1980s, endovascular embolization has become the standard of care in the management of VGAM. Modern techniques and materials allow staged occlusion of the arterial feeders using advanced microcatheters and liquid embolic systems and/or coils with the goal of complete treatment of the malformation. Yet, the high and turbulent flow characterizing the AV shunts in VGAM makes the procedure very challenging for the risk of glue migration and severe embolic complications (2). To increase the safety and efficacy of endovascular treatment, several adjunctive flow-control techniques have been developed to improve the accuracy of the treatment and prevent complications. Baranoski et al. in “Rapid ventricular overdrive pacing and other advanced flow-control techniques for the endovascular embolization of vein of Galen malformations” presented detailed flow-control techniques, including regionally targeted strategies (transvenous embolization and balloon-assisted transarterial embolization) and global flow-control methods (pharmacologic cardiac arrest and rapid ventricular overdrive pacing). The technical challenges and significant advancements in this field are presented in this review, highlighting the key role of the neurointerventional radiologist in the multidisciplinary management of this complex condition.

The identification of factors influencing neurological outcome is one of the main topics in VGAM research, with the aim of preventing acute and chronic neurological damage. Several aspects have been studied in cohort studies and meta-analyses (3–8), but it is very difficult to define clear-cut modifiable risk factors due to the rarity and extreme complexity of the disease. This problem is well demonstrated in the manuscript “Outcomes of endovascular embolization for Vein of Galen malformations: An individual participant data meta-analysis”. The goal of this study by Savage et al. was to identify risk factors associated with all-cause mortality and clinical outcome after endovascular embolization. Overall all-cause mortality was 16%, and overall good clinical outcome was achieved in 68% of participants. First embolization as a neonate, incomplete embolization, and heart failure at presentation were associated with the study outcomes. Despite the methodology (meta-analysis), and inclusion of older studies, this manuscript provides important data regarding risk factors and burden of disease in terms of mortality and morbidity.

Risk stratification, patient selection and time of intervention have a paramount role in the therapeutic planning. The case report by Saliou et al. “Pseudo-feeders as a red flag for impending or ongoing severe brain damage in vein of Galen aneurysmal malformation” is very illustrative. The authors highlight relevant arguments: the rapid onset and progression of cerebral damage in an infant with previously normal neuroimaging, the role of a specific factor in identifying at risk patients, and the importance of the appropriate time window for treatment. In particular, Saliou et al. focused on the role of arterial pseudofeeders as red flag of severe vascular steal phenomena through the shunt, and poor cerebral haemodynamic status.

A model of patient evaluation and therapeutic approach is presented in “Vein of Galen aneurysmal malformation in newborns: a retrospective study to describe a paradigm of treatment and identify risk factors of adverse outcome in a referral center,” by Buratti et al. In this single-center retrospective cohort study, the association of fetal and neonatal cardiologic and neuroradiologic parameters with severe high output heart failure (HOHF), endovascular complications and death in 40 consecutive newborns was assessed. Despite a high percentage of newborns developed severe haemodynamic compromise, none of the patients died due to HOHF and multiorgan failure. Specialized intensive care management focused on the complexity of VGAM pathophysiology and early endovascular treatment allowed reduction in mortality and optimization of clinical outcomes. Another relevant argument highlighted in this study is that severe congenital brain damage was the only indication for palliation, excluding critical hemodynamic compromise as a ground for palliation.

Campi et al., in “Neurodevelopmental and genetic findings in neonates with intracranial arteriovenous shunts: A case series” observed the importance of multimodal neurophysiological studies during the neonatal period. This retrospective observational study highlighted, despite inhomogeneities in the neurophysiological data, the value of early neurophysiological monitoring with aEEG and video EEG in the assessment of neurological status and in the early detection of complications secondary to the disease or to the endovascular treatment. Moreover, specific neurophysiological tests, in particular somatosensory evoked potentials, have been identified as promising potential markers of early and long term neurological impairment.

## Conclusions

It is well known that research on rare diseases is well-grounded and accurate if based on large scale data, international collaboration, and multidisciplinary debate. This editorial project launched by Frontiers two years ago created the opportunity for the international community to join in a shared research effort that went far beyond the initial aims. The VGAM network, developed thanks to the Frontiers Research Topic contributors, realized two relevant objectives: the first international meeting on VGAM in newborns and children held in Genoa, in May 2023, and the ongoing development of an international clinical research registry. We expect that these achievements, and new diagnostic and therapeutic options, such as endovascular liquid biopsy to study somatic mutations (9), ultrasound-guided fetal embolization (10), and gamma knife treatment in children (11) may define new perspectives and frame further promises for patients and families.
